# Association of C‐reactive protein and vitamin D deficiency with cardiovascular disease: A nationwide cross‐sectional study from National Health and Nutrition Examination Survey 2007 to 2008

**DOI:** 10.1002/clc.23189

**Published:** 2019-04-30

**Authors:** Qian Li, Zhenguo Dai, Yuze Cao, Lihua Wang

**Affiliations:** ^1^ Department of Neurology, The Second Affiliated Hospital Harbin Medical University Harbin China; ^2^ Department of Cardiology The Second Affiliated Hospital, Harbin Medical University Harbin China

**Keywords:** cardiovascular disease, C‐reactive protein, NHANES, vitamin D

## Abstract

**Objectives:**

The association of C‐reactive protein (CRP) and serum 25‐hydroxyvitamin D [25(OH)D] and cardiovascular disease (CVD) remains unknown.

**Methods:**

We performed a cross‐sectional analysis on 3848 participants by using the data from the National Health and Nutrition Examination Surveys (2007 to 2008). CVD was defined as a compromise of stroke, myocardial infarction, heart failure, and coronary heart disease. High CRP was defined as ≥0.2 mg/dL, and vitamin D status were categorized as severe deficiency, <25 nmol/mL; deficiency, 25 to 49.9 nmol/mL; insufficiency, 50 to 74.9 nmol/mL; and normal, ≥75 nmol/mL. Statistical analysis was performed using logistic regression models.

**Results:**

We found that both high CRP and low 25(OH)D levels were associated with CVD. Participants with high CRP levels and severe vitamin D deficiency had a higher likelihood of having CVD than those with neither risk factor (odds ratio = 2.69, 95% confidence interval = 1.45‐4.98, *P* = .0017). In stratified analysis, a significant positive association between vitamin D level and CVD was observed only in the high CRP group. However, in the absence of high CRP, even with severe vitamin D deficiency, no association was found with an increasing risk of CVD (*P* = .6416).

**Conclusion:**

Within a cross‐sectional, nationally representative sample, these findings suggest that vitamin D status evaluation, or vitamin D supplement may be especially important for individuals with high CRP levels.

## 1 INTRODUCTION

Cardiovascular disease (CVD) is considered traditionally as a major health concern that contributes to morbidity and mortality. Extensive and growing evidence suggested that inflammation is also a characteristic of this chronic process.^1^ Vitamin D is a fat‐soluble steroid, which has been demonstrated to play a key role in modulating inflammatory cytokines and regulating immune cell activity.^2^, ^3^ Emerging studies have indicated that vitamin D deficiency is linked to more inflammation and immune activation^4^ and can inversely be associated with a number of disease processes, including myocardial infarction.

C‐reactive protein (CRP), a sensitive marker of inflammation, has been shown to be implicated in predicting incident CVD in multiple prospective epidemiological studies.^5^ One study suggested that the addition of measurement of CRP to screening based on lipid levels may provide an improved method of identifying women at risk for cardiovascular events.^6^ It is well documented that some of the CVDs that were benefit from medical treatment and lifestyle modification (regular physical activity, diet), are resulting from attenuation in inflammation.^1^, ^7^, ^8^ Observational studies showed an association between low serum vitamin D levels and increased cardiovascular risk,^9^ while vitamin D sufficiency appeared to have favorable effects on CVD,^10^, ^11^ which may be partly associated with the immunomodulatory and anti‐inflammatory properties of vitamin D. Vitamin D supplementation is found to be associated with a reduction in inflammatory marker levels in patients with osteoporosis^12^, heart failure^13^, but not in healthy persons.^14^ Randomized controlled trials (RCT) and evidence regarding whether vitamin D intake from foods or supplements prospectively associated with lower CVD risk have shown inconsistent results, with some trials suggesting a benefit and others concluding no effect on CVD.^15^, ^16^, ^17^, ^18^, ^19^, ^20^, ^21^ There is possibility that the effect of vitamin D supplementation may vary by the status of CRP.

Therefore, in the present study, we tested the hypothesis of the combined association between 25(OH)D and CRP levels and CVD by using a representative sample of U.S. adults participating in the National Health and Nutrition Examination Survey (NHANES) 2007 to 2008.

## 2 METHODS

### 2.1 Data source and study population

We used data from NHANES conducted by the National Center for Health Statistics (NCHS), Centers for Disease Control and Prevention (CDC). Informed consent was obtained from all participants. The study is approved by the institutional review board of the National Center for Health Statistics. Data were pooled the cross‐sectional data from the 2007 to 2008 NHANES cycle. In NHANES self‐report data, participants were considered to have a history of cardiovascular disease if they responded “yes” to the question, “Has a doctor ever told you that you had congestive heart failure or coronary heart disease or heart attack or stroke?” CVD was defined as a compromise of stroke, myocardial infarction, heart failure, and coronary heart disease. The total population of NHANES 2007 to 2008 was 10 149. Participants were selected between 20 and 80 years of age (4464). Of the 4464 eligible subjects, 3848 had no missing data for all relevant covariates required for the analysis.

### 2.2 Serum 25(OH)D and CRP concentration

For serum 25(OH)D measurements, the approaches transited to the liquid chromatography‐tandem MS (LC‐MS/MS) measurement procedure since NHANES 2007 and 2008. In addition, the LC‐MS/MS allows for separate quantification of serum 25‐hydroxyvitamin D_2_ [25(OH)D_2_] (Isosciences, King of Prussia, Pennsylvania; Sigma, St. Louis, Missouri) and 25(OH)D_3_ (USP, Rockville, Maryland; Sigma).^22^ Serum 25(OH)D levels were then categorized as most of the previous clinical studies suggested, normal values (≥75 nmol/mL), insufficient (50‐74.9 nmol/mL), deficient (25‐49.9 nmol/mL), and severely deficient (<25 nmol/mL).^23^, ^24^ Serum CRP was measured as described in the NHANES Laboratory Procedures Manual. CRP was measured using latex‐enhanced nephelometry (Dade Behring Diagnostics Inc., Somerville, New Jersey), with high CRP levels defined as 0.20 mg/dL or greater. This process was then repeated substituting vitamin D deficiency for severe vitamin D deficiency referred to previous literature.^25^

### 2.3 Covariates

We chose covariates as potential confounding factors based on prior studies or based on their biological plausibility. The following covariates were included in our association analysis: age, sex, race/ethnicity, body mass index (BMI), family poverty income ratio (PIR), marital status, educational level, smoking status, alcohol consumption, physical activity, hypertension, diabetes, hypercholesterolemia, chronic kidney disease (CKD), and season of blood collection. Race/ethnicity was classified as non‐Hispanic white, non‐Hispanic black, Mexican American, and other. Marital status was classified as single/never married, married/live together, widowed, separated/divorced, and others. We categorized educational level as less than a high school education/primary education, a high school education/secondary education, and a college education. BMI was calculated as weight in kilograms divided by height in meters squared (kg/m^2^). Height and weight were collected in the mobile examination center. The self‐reported PIR, a marker of socioeconomic status used in multiple prior studies.^26^ Smoking status was classified as never, former, or current, which was constructed from responses to two questions: “Have you smoked at least 100 cigarettes in your life?” and “Do you now smoke cigarettes?” Participants who reported smoking every day or some days and had smoked at least 100 cigarettes were categorized as current smokers; respondents who reported currently not smoking but having smoked more than 100 cigarettes in the past were categorized as former smokers; and respondents who reported having smoked fewer than 100 cigarettes ever were categorized as non‐smokers.^27^ Alcohol consumption was defined as having at least 12 drinks of any type of alcoholic beverage in the past year.^28^ For physical activity, the weekly number of minutes of moderate or vigorous activity, and the weekly number of minutes of vigorous activity were summed. Ideal health (active) was defined as ≥150 minutes/week of moderate or vigorous activity; intermediate health was defined as 1 to 149 minutes/week of moderate or vigorous activity; and poor health (inactive) was defined as 0 minute/week of moderate or vigorous activity.^28^ Hypertension was diagnosed if the participants reported being told by a physician that they have high blood pressure, were taking antihypertensive medications, or the average of three blood pressure readings was ≥140/90 mmHg.^28^ Hypercholesterolemia was diagnosed if total serum cholesterol ≥240 mg/dL or on cholesterol medications.^28^ Participants were defined as having diabetes when a physician had ever told them that they had diabetes, excluding during pregnancy, with concurrent use of insulin or oral hypoglycemic medication, or a hemoglobin A1c ≥ 6.5%, non‐fasting glucose ≥200 mg/dL, or fasting glucose ≥126 mg/dL.^29^ Dates of blood collections performed at physical examination visits were recorded and operationalized as November 1 to April 30 or May 1 to October 31. Glomerular filtration rate (GFR) was estimated based on the Chronic Kidney Disease Epidemiology Collaboration (CKDEPI) equation CKD‐EPI Equation.^30^ In these analyses, CKD was defined as estimated GFR less than 60 mL/min/1.73 m^2.^ 31

## 3 STATISTICAL ANALYSIS

All analysis were performed with R (http://www.R-project.org). Differences in participant characteristics according to whether they had CVD were compared using independent‐samples *t* tests (normal distribution) or Kruskal‐Wallis rank sum test (non‐normal distribution) for continuous measurements and χ² tests for categorical data or measurements.

Multiple logistic regression was used to determine the independent association between 25(OH)D status, with normal 25(OH)D as the reference category, and high and low CRP levels and CVD, respectively, after adjustment for demographic variables including age, sex, race/ethnicity, physical activity, educational level, marital status, family PIR, CKD, alcohol consumption, BMI, hypertension, diabetes mellitus, hypercholesterolemia, and season of specimen collection (6‐month period). Two additional logistic regression models were used to evaluate the association of vitamin D and CRP with CVD after controlling for the same confounders.

Finally, logistic regression was used to evaluate the association between vitamin D status and CVD after stratifying participants according to high and low CRP status. The association between CRP status and CVD was also analyzed using logistic regression after stratification according to vitamin D status (severe deficiency, deficiency, insufficiency, normal) and their interactions were tested. A *P*‐value of <.05 was considered statistically significant.

## 4 RESULTS

Of the 10 149 eligible subjects from NHANES (2007‐2008), 3848 had no missing data for all relevant covariates required for the analyses (n = 3439 in non‐CVD, n = 409 in CVD). The baseline characteristics of the subjects in the analytic sample were separated by CVD and non‐CVD were shown in Table 1. The percentage of subjects with CVD was higher in those with lower levels of vitamin D, yet this did not reach a statistically significant (*P* = .887). Participants with CVD had a significantly greater frequency of high CRP than those without (64.06% vs 50.10%; *P* < .001). On average, individuals with CVD were more likely to be older, male, non‐Hispanic black, a former smoker, less educated, and less physically active than participants without CVD. Moreover, they had higher BMI, lower family PIR; they were less likely to be married and non‐smokers, and had more traditional CVD risk factors, such as hypertension, diabetes mellitus, hypercholesterolemia, and CKD (Table 1).

**Table 1:** Characteristics of participants between 20 and 80 years of age stratified by cardiovascular disease (NHANES 2007‐2008)

	No CVD	CVD	*P*‐value
Number	3439	409	
25(OH)D (nmol/mL)	(62.24 ± 25.22)	60.73 ± 24.45	.395
Normal (**≥**75)	978 (28.44)	114 (27.87)	.887
Insufficiency (50‐74.9)	1294 (37.63)	152 (37.16)	
Deficiency (25‐49.9)	996 (28.96)	119 (29.10)	
Severe deficient (<25)	171 (4.97)	24 (5.87)	
CRP (mg/dL)	0.43 ± (0.81)	0.66 ± 1.34	<.001
<0.2	1716 (49.90)	147 (35.94)	<.001
**≥**0.2	1723 (50.10)	262 (64.06)	
Age, year (means ± SD)	48.65 ± 17.27	66.97 ± 12.29	<.001
Sex, males, n (%)	1644 (47.80)	250 (61.12)	<.001
Race/ethnicity, Black, n (%)	1666 (48.44)	257 (62.84)	<.001
BMI (kg/m^2^), (means ± SD)	28.94 ± 6.57	30.18 ± 7.00	<.001
Family PIR, (means ± SD)	2.60 ± 1.63	2.28 ± 1.47	<.001
Marital status, n (%)			<.001
Married	2125 (61.79)	236 (57.70)	
Widowed	239 (6.95)	82 (20.05)	
Divorced/seperated	503 (14.63)	58 (14.18)	
Single	572 (16.63)	33 (8.07)	
Educational level, n (%)			<.001
Less than a high school education/primary education	994 (28.90)	154 (37.65)	
High school education/secondary education	832 (24.19)	98 (23.96)	
College education	1613 (46.90)	157 (38.39)	
Smoking status, n (%)			<.001
Current smoking	756 (21.98)	84 (20.54)	
Former smoking	812 (23.61)	179 (43.77)	
Never smoking	1871 (54.41)	146 (35.70)	
Alcohol consumption, n (%)	2439 (70.92)	276 (67.48)	.235
Physical activity (minutes/week)			<.001
No (0)	1891 (54.99)	282 (68.95)	
Inactve (1‐149)	255 (7.41)	24 (5.87)	
Active (≥150)	1293 (37.60)	103 (25.18)	
Hypertension, n (%)	976 (28.38)	297 (72.62)	<.001
Diabetes mellitus, n (%)	308 (8.96)	129 (31.54)	<.001
Hypercholesterolemia, n (%)	1057 (30.74)	250 (61.12)	<.001
CKD, n (%)	261 (7.59)	122 (29.83)	<.001
Season of blood collection, n (%)			<.001
November 1 to April 30	1526 (44.37)	136 (33.25)	
May 1 to October 31	1913 (55.63)	273 (66.75)	

Abbreviations: BMI, body mass index; CKD, chronic kidney disease; CRP, C‐reactive protein; CVD, cardiovascular disease; PIR, poverty income ratio; 25(OH)D, 25‐hydroxyvitamin D.

Values are presented as mean ± SD or number, (percentage of distribution).

Table 2 shows the OR of cardiovascular events according to the level of 25(OH)D and CRP. Subjects with severe vitamin D deficiency were more likely to have CVD (odds ratio [OR] = 1.90, 95% confidence interval [CI] = 1.06, 3.40, *P* = .0317). Those with insufficient vitamin D or vitamin D deficiency were also more likely to have CVD, yet these findings did not reach statistical significance (*P* > .05). When 25(OH)D level was entered into the model as a pseudo‐continuous variable, the likelihood of having CVD increased with decreasing levels of 25(OH)D (*P* = .0128). Participants with high CRP had a significantly increased chance to have CVD than those with low CRP (OR = 1.47, 95% CI = 1.14‐1.89, *P* = .0029).

**Table 2:** Odds ratios for cardiovascular disease, based on vitamin D status and CRP status in National Health and Nutritional Examination Survey 2007 to 2008 (N = 3848)

	OR (95% CI)	*P*‐value
Vitamin D status (nmol/mL)	1.21 (1.04, 1.41)	.0128
Normal (≥75) (n = 1092)	1.00	
Insufficiency (50‐74.9) (n = 1446)	1.11 (0.83, 1.50)	.4840
Deficiency (25‐49.9) (n = 1115)	1.40 (1.00, 1.96)	.0514
Severe deficient (<25) (n = 195)	1.90 (1.06, 3.40)	.0317
High CRP (≥0.2 mg/dL) (n = 1985)	1.47 (1.14, 1.89)	.0029

Abbreviations: CI, confidence interval; CRP, C‐reactive protein; OR, odds ratio.

Model adjusted for age, sex, race, physical activity, educational level, marital status, family poverty income ratio, body mass index, season of blood collection, smoking status, and alcohol drinking, hypertension, diabetes mellitus, hypercholesterolemia, and history of chronic kidney desease.

Table 3 shows the results from the logistic regression analyses evaluating the association between vitamin D, CRP, and CVD. Combination of severe vitamin D deficiency and high CRP was associated with a higher chance to have CVD (OR = 2.69, 95% CI = 1.45‐4.98, *P* = .0017). In contrast, there was no significant evidence that having severe vitamin D deficiency without high CRP was associated with increasing likelihood of CVD (*P* > .05), but participants with high CRP without severe vitamin D deficiency were more likely to have CVD (OR = 1.42, 95% CI = 1.10‐1.84, *P* = .0068). Similar combined results were found in participants with vitamin D deficiency and high CRP (OR = 2.01, 95% CI = 1.39‐2.89, *P* = .0002); while having vitamin D deficiency and low CRP did not show significant association with CVD.

**Table 3:** Association between vitamin D level, CRP level and cardiovascular disease in National Health and Nutritional Examination Survey 2007 to 2008 (N = 3848)

Effect	n	OR (95% CI)	*P*‐value
Combination of vitamin D level (deficiency/no deficiency) and CRP level (low/high)			.0006
No vitamin D deficiency and low CRP	1314	1.00	
Vitamin D deficiency and low CRP	549	1.19 (0.77, 1.86)	.4365
No vitamin D deficiency and high CRP	1224	1.38 (1.02, 1.86)	.0360
Vitamin D deficiency and high CRP	761	2.01 (1.39, 2.89)	.0002
Combination of vitamin D level (severe deficiency/no severe deficiency) and CRP level (low/high)			.0008
No severe vitamin D deficiency and low CRP	1790	1.00	
Severe vitamin D deficiency and low CRP	73	0.74 (0.21, 2.63)	.6416
No severe vitamin D deficiency and high CRP	1863	1.42 (1.10, 1.84)	.0068
Severe vitamin D deficiency and high CRP	122	2.69(1.45, 4.98)	.0017

Abbreviations: BMI, body mass index; CI, confidence interval; CRP, C‐reactive protein; OR, odds ratio; 25(OH)D, 25‐hydroxyvitamin D.

Vitamin D deficiency: <50 nmol/mL; No vitamin D deficiency: ≥50 nmol/mL; Severe vitamin D deficiency: <25 nmol/mL; No severe vitamin D: ≥25 nmol/mL; low CRP: <0.2 mg/dL; and high CRP: ≥0.2 mg/dL.

Model adjusted for age, sex, race, physical activity, educational level, marital status, family PIR, body mass index, season of blood collection, smoking status, and alcohol drinking, hypertension, diabetes mellitus, hypercholesterolemia, history of chronic kidney desease.

We then evaluated the association between vitamin D and CVD based on high or low CRP level. The ORs for CVD associated with 25(OH)D level are listed in Table 4 and were different between different CPR level status (high or low). We observed a significant association between vitamin D level and CVD only in the high CRP group, but not in the low CRP group across all three logistic regression models. In subjects with high CRP, the OR of having CVD was 2.43 (95% CI = 1.23‐4.82, *P* = .0110) in individuals who also exhibited severe vitamin D deficiency compared with those with normal vitamin D levels. Those with lower 25(OH)D levels were more likely to have had a CVD outcome, and was dependent of CRP status (Table 4).

**Table 4:** Cross‐sectional association between vitamin D level and cardiovascular disease, according to CRP level, in the National Health and Nutritional Examination Survey 2007 to 2008 (n = 3848)

Vitamin D status (nmol/mL)	Model 1		Model 2		Model 3	
	OR (95% CI)	*P*‐value	OR (95%CI)	*P*‐value	OR (95% CI)	*P*‐value
CRP ≥ 0.2 mg/dL (n = 1985)						
Normal (≥75) (n = 495)	1.00		1.00		1.00	
Insufficiency (50‐74.9) (n = 729)	1.20 (0.83, 1.74)	.3394	1.09 (0.75, 1.60)	.6431	1.15 (0.78, 1.72)	.4803
Deficiency (25–49.9) (n = 639)	1.67 (1.12, 2.49)	.0122	1.37 (0.91, 2.08)	.1332	1.45 (0.94, 2.24)	.0908
Severe deficient (<25) (n = 122)	2.42 (1.28,4.56)	.0063	1.90 (0.99, 3.66)	.0533	2.43 (1.23, 4.82)	.0110
CRP < 0.2 mg/dL (n = 1863)						
Normal (≥75) (n = 597)	1.00		1.00		1.00	
Insufficiency (50–74.9) (n = 717)	1.10 (0.71, 1.69)	.6812	1.02 (0.65, 1.59)	.9400	1.02 (0.64, 1.61)	.9457
Deficiency (25–49.9) (n = 476)	1.45 (0.86, 2.45)	.1587	1.28 (0.75, 2.21)	.3658	1.29 (0.74, 2.28)	.3717
Severe deficient (<25) (n = 73)	1.00 (0.27, 3.70)	.9963	0.87 (0.23, 3.31)	.8347	0.80 (0.20, 3.16)	.7541

Abbreviations: CI, confidence interval; CRP, C‐reactive protein; OR, odds ratio.

Model 1: adjusted for age, gender, and race at baseline.

Model 2: adjusted for variables in model 1 and physical activity, educational level, marital status, family poverty income ratio, body mass index, season of blood collection, smoking status, and alcohol drinking.

Model 3: adjusted for variables in model 2 and hypertension, diabetes mellitus, hypercholesterolemia, history of chronic kidney desease.

## 5 DISCUSSION

In the present study, we performed a large cross‐sectional study of nationally representative sample of adults >20 years of age by using continuous NHANES (2007‐2008). In addition to being a disease of modified lipoproteins accumulation in the artery wall, inflammation is also an additional key aspect of this disease process.^32^ In this study, we chose CRP as such a marker of inflammation, and confirmed that individuals with high CRP had a higher likelihood of CVD (Table 2). Lower vitamin D status was previously found to be associated with increased risk and unfavorable outcome of CVD,^33^, ^34^ herein, our results revealed that individuals with both high CRP and severe vitamin D deficiency had 2.69 times the likelihood for experiencing CVD, which was higher than those with neither of them (Table 3). In terms of clinical application, the addition of the measurement of CRP to screening based on vitamin D levels may thus provide a simple and inexpensive method to improve risk prediction for cardiovascular events.

In the stratified analysis of the current study, we found that in the absence of inflammation, even having severe vitamin D deficiency was not associated with increasing likelihood of CVD, but participants with high CRP without severe vitamin D deficiency were still more likely to have CVD (Table 3). The findings indicate that the link between vitamin D and CVD depends on the inflammation status. Thus, recommendations to improve vitamin D status on the basis of monitoring CRP levels may be of benefit to reduce the burden of CVD. Additional well‐designed clinical studies may be warranted to determine whether individuals with high CRP levels are especially important for vitamin D to exert the protective effect against CVD.

There are evidence supporting the potential association of vitamin D and CRP with CVD observed in this study. Inflammatory state manifested by high CRP levels may lead to low 25(OH)D levels, and reduction of vitamin D contributes to the dysregulation of immune and inflammatory responses, such as respiratory infections and psoriasis.^3^, ^35^, ^36^ Moreover, studies in animal models and cell culture showed that vitamin D treatment could arrest nuclear factor κB translocation and suppress its activity,^37^ and could produce anti‐inflammatory effects by modulating immune cells and signaling pathways.^3^

Although the present study is a cross‐sectional observational study, our results have the advantage of containing a large and nationally representative sample size to better estimate association of high CRP and low vitamin D with CVD, giving credence to the external validity of the results. Moreover, the NHANES allowed us to adjust for numerous potential confounders in the analysis. Furthermore, serum 25(OH)D concentrations were measured using a LC‐MS/MS measurement procedure developed by the CDC, which is considered more analytically accurate, precise, and specific than immunoassay methods.^22^

However, in interpreting the results of this study, some limitations in the present study still exist due to its intrinsic cross‐sectional nature. First of all, for the sake of data integrity, we only studied one NHANES cycle from 2007 to 2008. Second, the information selected on vitamin D, CRP, and CVD was collected at the same point, our study could not ensure to examine a temporal relation between vitamin D and CRP. Third, the study is unable to test the risk of decreased serum vitamin D and/or increased CRP concentrations for the incidence of CVD. At last, data on the diets enriched with vitamin D, including supplements, that could modify 25(OH)D blood levels were not available for analysis.

## 6 CONCLUSIONS

In conclusion, although our study is a cross‐sectional study, we believe that our findings are of great public health significance. Subjects with vitamin D deficiency and CRP levels ≥0.2 mg/dL represent a higher chance to have CVD. Vitamin D was significantly associated with CVD only in those with high CRP, this statistical evidence supports the view that the burden of CVD in individuals with high CRP may be reduced by improving serum 25(OH)D concentrations. Future RCTs should focus on the effects of vitamin D supplements on CVD by comparing individuals with high CRP and those with low CRP.

## CONFLICT OF INTEREST

The authors declare no potential conflict of interests.
